# Intraosseous angiolipoma of the humerus: a second case report

**DOI:** 10.1097/MS9.0000000000000737

**Published:** 2023-05-08

**Authors:** Firas K. Khana, Sina Khabbak, Sana Shaikh Torab, Mohammed W. Arslan, Mohamed I.E. Mouhandes

**Affiliations:** a Department of Medical Imaging and Diagnostic Radiology, Aleppo University Hospital (AUH); b Private Practice Pathologist, Aleppo, Syria

**Keywords:** case report, fat suppression, humerus, intraosseous angiolipoma, magnetic resonance imaging

## Abstract

To document a case of an intraosseous angiolipoma (IOAL) of the humerus.

**Case presentation:**

A 52-year-old woman presented with gradually increasing pain in her right arm. A X-ray series of the right elbow and computed tomography revealed a nonexpanding radiolucent lesion in the distal end of the right humerus. The fatty nature of the lesion was further verified using fat-suppression techniques on magnetic resonance imaging sequences.

**Clinical discussion:**

The tumor was surgically excised, and the patient has experienced no symptoms for 2 years now. Histopathological findings confirmed the presence of an IOAL, which is an extremely rare intraosseous tumor that consists of thin blood vessels and mature adipose tissue.

**Conclusion:**

Accurate diagnosis of an IOAL is challenging; therefore, careful planning and assessment are paramount in the management of such lesions, with histological findings being essential for a conclusive diagnosis and surgery is the most suitable treatment choice in most cases.

## Introduction and importance

Highlights

- Intraosseous angiolipomas of the humerus are difficult to diagnose.
- They are often asymptomatic but may cause pain if they grow large enough.
- MRI can verify the presence of fat inside the lesion.
- Histopathological findings are crucial to diagnosis.
- Treatment typically involves surgical excision or embolization.

Angiolipomas are benign soft tissue tumors characterized by their slow growth and unique histological components comprising both mature adipose tissue admixed with abnormally thin blood vessels^1^.

They are considered relatively common, generally occurring as subcutaneous lesions termed subcutaneous angiolipomas, and can occur anywhere in the body, more commonly so in the trunk and the proximal upper limbs. Intraosseous angiolipomas (IOALs) are considered extremely rare, on the other hand, occurring mostly in the ribs and mandible.

Being an extremely rare finding, extreme caution must be taken to avoid confusion with lesions that can mimic their clinical and radiological appearance on various imaging modalities, including lytic bone lesions such as aneurysmal bone cysts, lipomas, angiomas, hemangiomas, giant cell tumors, fibrous dysplasia, and even meningiomas^2–4^.

The aim of this study is to document a rare case of an IOAL occurring in the distal end of the right humerus. Based on what we know, this is the second case of its kind and the twenty-first case of an IOAL reported in the literature.

This case report has been reported in line with the SCARE (Surgical CAse REport) criteria^5^.

## Case presentation

A 52-year-old Caucasian woman presented with a chief complaint of gradually increasing pain in the right elbow. The pain started as a mild discomfort in the elbow region and eventually increased in severity over the course of 1 year. Physical examination revealed moderate localized tenderness in the distal end of the right humerus that did not spread to other regions. She also complained of hypertension which was well-regulated with medications. She was nonalcoholic and had no previous family or allergy history.

Upon performing laboratory investigations, an increased level of white blood cell count was noted (12 000 WBCs per microliter), with the rest of the tests yielding normal results.

**Figure 1:**
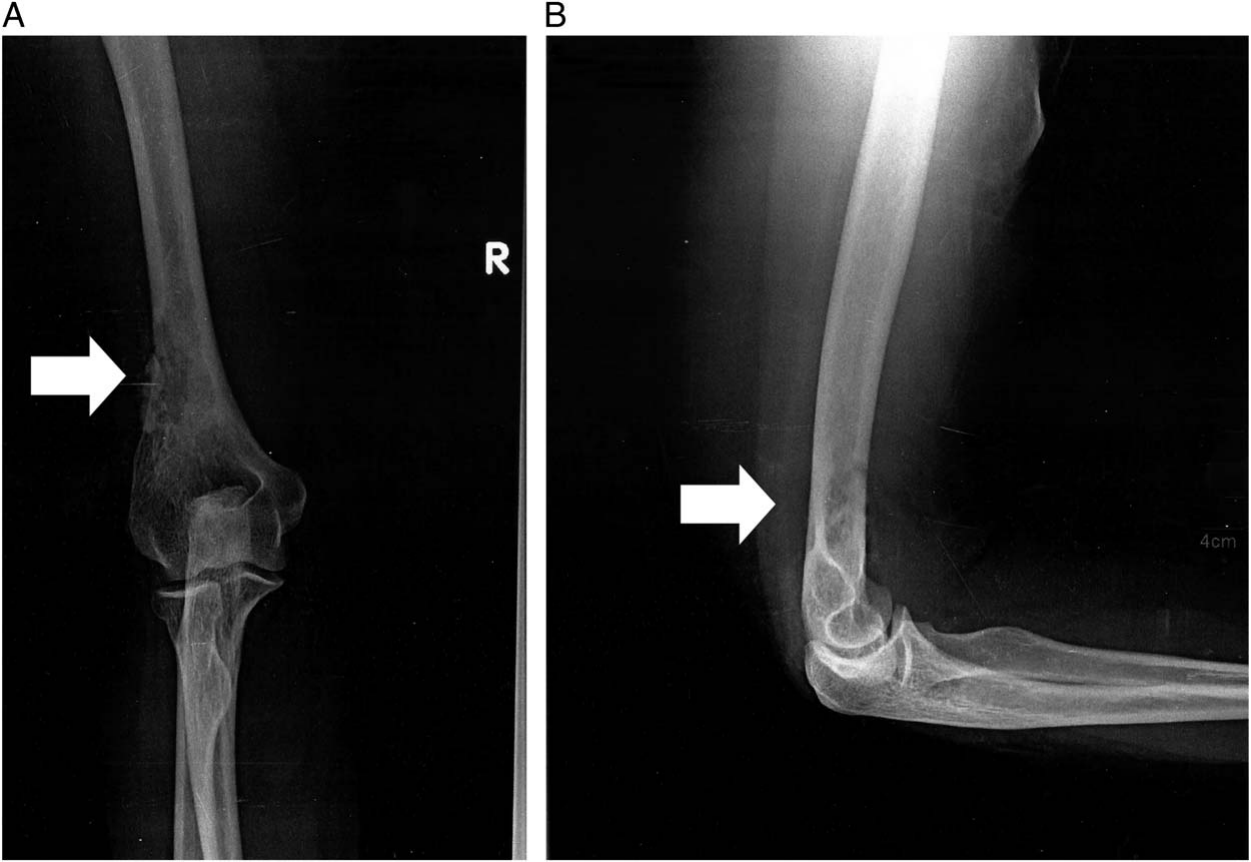
Anteroposterior (A) and lateral (B) projections of the elbow demonstrating a nonexpanding radiolucent lesion in the distal humerus (white arrow).

An elbow series with both AP and lateral projections was ordered and revealed a well-defined, nonexpanding radiolucent lesion in the distal end of the humerus, as shown in Figure 1A, B.

**Figure 2:**
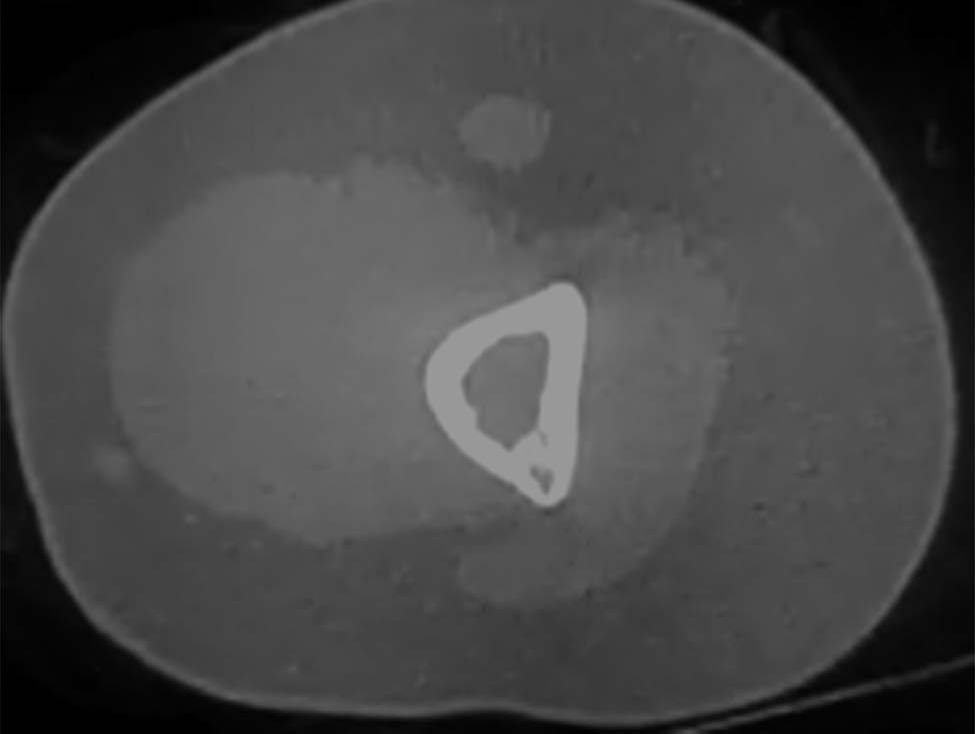
Axial view of the distal humerus showing an osteolytic lesion with fatty content.

A noncontrast-enhanced computed tomography showed a nonexpanding osteolytic lesion with fatty tissue inside, also depicted in Figure 2.

**Figure 3:**
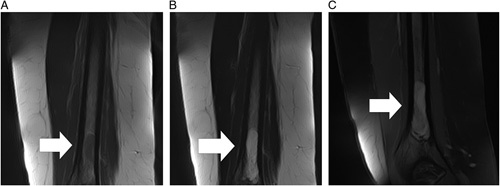
Coronal planes show an isointense to hypointense mass on unenhanced T1-weighted images (A) and isointense to hyperintense on T2-weighted images (B), with the enhancement of the vascular component on gadolinium-enhanced fat-saturated T1-weighted images (C), located in the distal third of the humerus (white arrow).

Contrast-enhanced magnetic resonance imaging (CE-MRI) of the distal humerus showed a well-circumscribed iso-to-hypointense lesion on T1-weighted images with distinct margins. The lesion appeared hyperintense on T2-weighted images and isointense on PD-weighted images with the signal from the fatty tissue inside being moderately suppressed on sequences utilizing fat-suppression techniques [e.g. Fat-Sat (Fat Saturation) and STIR (Short Tau Inversion Recovery)], which helped identify its fatty composition. The lesion demonstrated a heterogenous enhancement pattern with the pronounced enhancement of its vascular component post-contrast administration, as seen in Figure 3A–C.

**Figure 4:**
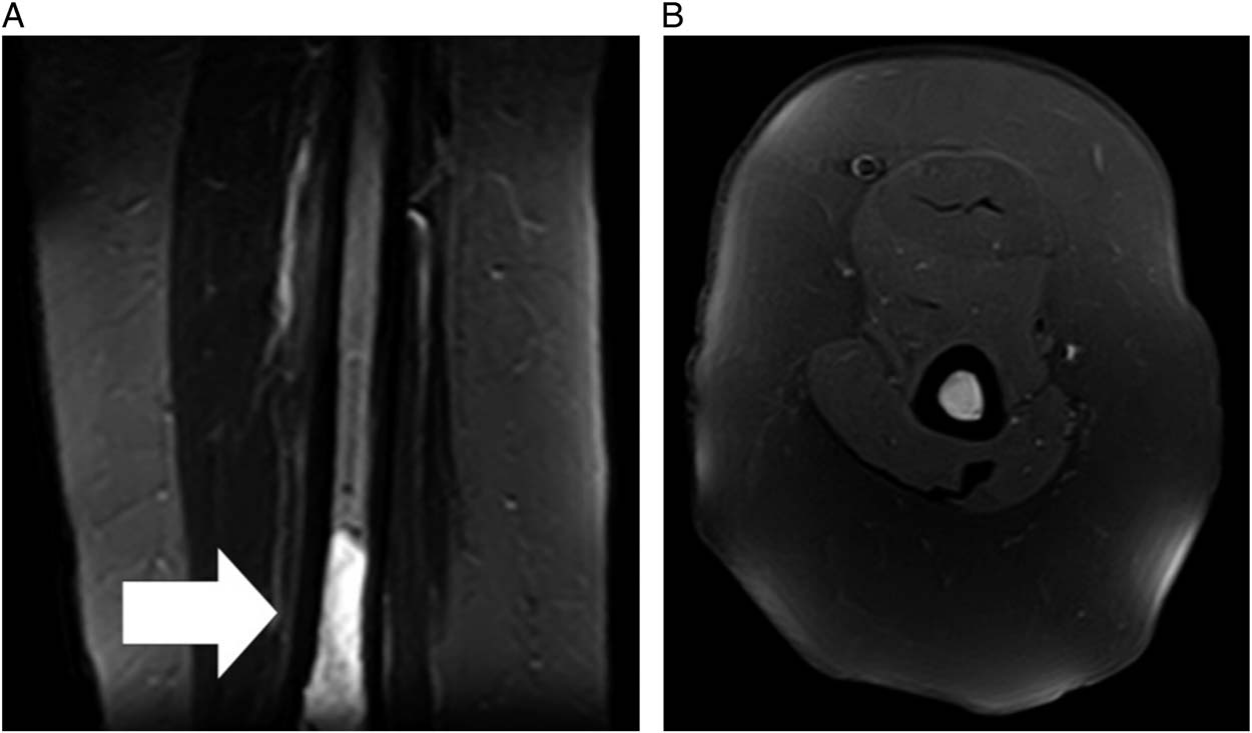
Coronal and axial planes illustrating the difference of fat-suppression techniques, comparing T2-weighted STIR image (A) against fat-saturated PD-weighted image (B).

Comparison of fat-suppression techniques as shown in Figure 4A, B.

The surrounding tissue was unremarkable on all previously mentioned imaging modalities, validating the noninfiltrative nature of the lesion.

**Figure 5:**
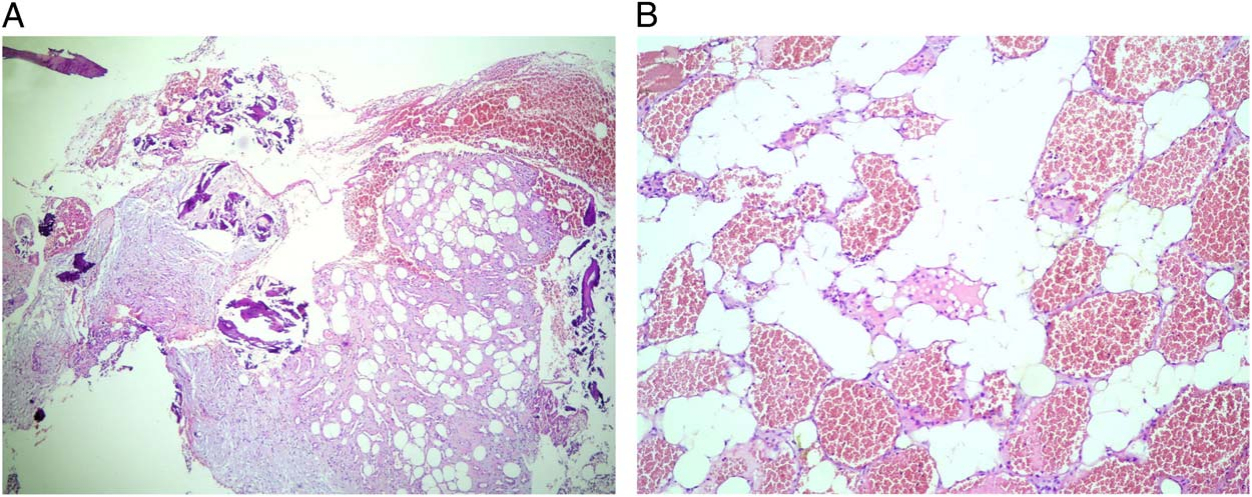
Microscopical images obtained during histological examination showing mature adipocytes surrounded by disorganized thin-walled blood vessels in an angiolipomatous configuration [hematoxylin and eosin (H&E) stain, original magnification ×40 (A) and ×100 (B)].

The humeral lesion was surgically resected by scraping the stiff bony tissue and some surrounding intricate soft tissue. The overall scrape measured 4 cm and was sent entirely to pathology. Histopathological findings revealed some septa of variable thickness composed of soft connective tissue and thin-walled blood vessels of variable diameters, with signs of old petechiae and hemosiderin depositions in addition to scattered infiltrations of lymphocytes and plasma cells. Thin bony septa were also seen on the margins of the previously mentioned tissues, along with vast islands of mature adipocytes, as seen in Figure 5. These findings were in line with the imaging findings and a final diagnosis of IOAL was reached.

## Clinical discussion

Having conducted a thorough study of the literature, we came to the conclusion that there were a total of 20 reported cases, as shown in Table 1 below, with 8 being cranial lesions^3,4,6–11^, 7 occurring in the mandible^12–18^, 3 in the ribs^19–21^, 1 in the calcaneus^22^, and 1 in the head of the humerus^2^, and our case, which we are positive is the second of its type to denote an IOAL involving the humerus, involving its distal end.

**Table 1:** Summary of all reported intraosseous angiolipoma cases in literature at the time of writing this article.

ID	Location	Age	Sex	Clinical presentation	History of trauma	Imaging findings	Provisional diagnosis
1	Left body of the mandible	39	M	Hyperesthesia of the left lower lip	None	X-ray: Bilateral well-circumscribed nonexpanding radiolucent lesions	Myxoma, lipoma
2	Left body of the mandible	56	F	Asymptomatic	Cyst removal 15 years ago	X-ray: Multilocular nonexpanding radiolucent lesion	Ameloblastoma, multilocular odontogenic cyst
3	Right third rib	27	M	Asymptomatic	N/A	X-ray: Well-defined expansile lesion	Fibrous dysplasia, aneurysmal bone cyst, giant cell tumor, enchondroma, eosinophilic granuloma, metastases, plasmacytoma
						CT: Ovoid lesion with Hounsfield number measurements ranging from −26 to +78	
4	Left foot	33	F	N/A	Minor trauma	X-ray: Round, well-circumscribed radiolucent lesion	Fibrous dysplasia, giant cell tumor, enchondroma, aneurysmal bone cyst, osteoblastoma, chondroblastoma, nonossifying fibroma, liposarcoma
5	Left ramus and posterior body of the mandible	51	F	Asymptomatic	None	X-ray: Well-defined mixed radiolucent and radiopaque lesion with cortical thickness	Hemangioma
						CT: Widening of the marrow space	
6	Rib	N/A	N/A	N/A	N/A	N/A	N/A
7	Right parietal bone of the cranium	50	M	Swelling	Minor trauma	CT: Large expansile lesion with bony spicules and midline shift to the left	Cranial angioma
						Cerebral angiography: Distinct hypervascular appearance with a persistently delayed blush	
8	Left body of the mandible	21	F	Swelling	None	X-ray: Expansile radiolucent lesion	Vascular malformation, hemangioma, central giant cell granuloma
						CT: Expansile lesion	
9	Left frontotemporoparietooccipital bones of the cranium	41	F	Swelling, tenderness, headache	None	X-ray: Wide expansile trabeculated radiolucent area	Bone cyst, hemangioma, eosinophilic granuloma, intraosseous meningioma, Paget’s disease, fibrous dysplasia, metastases
						CECT: Expansile nonenhancing lesion causing cerebral compression with Hounsfield number measurements ranging from −50 to +100	
						CE-MRI: Expansile nonenhancing hyperintense trabeculated lesion on T1W and T2W images with remarkable mass effect	
10	Right frontal bone of the cranium	55	M	Facial asymmetry, headache, nausea, vomiting, diplopia	N/A	CT: Expansile trabeculated lesion without soft tissue or brain involvement	Metastases from invasive ductal carcinoma of the right breast
						CE-MRI: Heterogeneously enhancing lesion with significant vascularity and mild mass effect	
11	Right frontal bone of the cranium	16	F	Swelling, headache	None	CT: Well-defined hypodense expansile lytic lesion, with no bone destruction and with a Hounsfield number measurement of -80	Lipoma, hemangioma, Langerhans histiocytosis, epidermoid cyst, metastases
12	Left fourth rib	46	M	Left-sided chest pain	N/A	X-ray: Lobulated area of increased density	N/A
						CT: Expansile lobulated lesion with fat density	
13	Right parietal bone of the cranium	30	F	Altered sensation without pain	None	CT: Expansile lesion with mass effect	N/A
						MRI: Expansion to extra-axial space causing cerebral compression without bone destruction	
14	Proximal end of the right humerus	17	M	Pain in the right shoulder	Humerus fractures, 2 years ago	X-ray: Lytic lesion with bony trabeculae and septations	N/A
						CT: Lytic lesion with fatty component, bony trabeculae, and septations	
15	Left frontoparietal bone of the cranium	61	F	Swelling	Minor trauma	CT: Expansile spiculated osseous lesion with small soft tissue component	Metastatic disease, atypical hemangioma
						CE-MRI: Expansile striated enhancing bone lesion with extension to the soft tissues	
						Bone scan: Solitary lesion in the skull	
16	Right body of the mandible	62	M	Asymptomatic	None	X-ray: Lytic lesion with bony trabeculae and septations	Periapical cyst, odontogenic, keratocyst, ameloblastoma
						CBCT: Well-circumscribed hypodense lesion with sclerotic margins and no bone resorption	
17	Left frontal and parietal bones of the cranium	50	F	Headache, nausea, vomiting	N/A	CT: Two expansile hypodense lesions with interior calcifications causing compression of cerebral parenchyma	N/A
						CE-MRI: Two hyperintense extra-axial lesions on T1W and T2W images with mild contrast enhancement	
18	Right ramus of the mandible	58	F	Asymptomatic	N/A	CBCT: Well-circumscribed multilocular hypodense lesion with scalloped borders	Vascular lesions, benign odontogenic entities, central giant cell granuloma, low-grade intraosseous malignancies
19	Right frontal bone of the cranium	72	F	Swelling, seizures, cognitive dysfunction	N/A	CECT: Expansile hypodense lesion with increased blood flow on perfusion imaging, causing compression of cerebral parenchyma	
						CE-MRI: Hyperintense lesion on T1W and T2W images, with marked fat suppression and heterogenous enhancement	
20	Right body of the mandible	66	M	Asymptomatic	N/A	CT: Well-defined radiolucent lesion	N/A
21	Distal end of the right humerus	52	F	Pain in the right elbow	None	X-ray: Well-defined, nonexpanding radiolucent lesion	Bone cyst, intraosseous ganglion cyst, hemangioma, lipoma
						CT: Nonexpanding osteolytic lesion with fatty content	
						CE-MRI: Iso-to-hypointense lesion on T1W images, and hyperintense on T2W, with moderate fat suppression and marked enhancement post-contrast administration	

CBCT, cone beam computed tomography; CECT, contrast-enhanced computed tomography; CE-MRI, contrast-enhanced magnetic resonance imaging; CT, computed tomography.

The clinical presentation varies greatly depending on the location of the lesions. The most frequently reported symptoms are pain and localized swelling, with one-third of patients being asymptomatic^11–14,16,21^. A predilection for women was noted, accounting for two-thirds of the reported cases^3,6,8–11,13–18,23^. The mean age of presentation is 43 years old.

The etiology of IOAL remains unclear, which prompted the introduction of many presumptive theories suggesting ‘trauma’ as a plausible cause. However, Hart *et al*. documented ‘trauma’ as the presumable cause in only four cases and concluded that its causation is somewhat unlikely^2,4,8,17^.

Ida-Yonimochi *et al*. noted the existence of mast cells in an angiolipoma occurring in the buccal muscle that were immunoreactive for a vascular endothelial growth factor (VEGF), signifying the important role of mast cells in the production of VEGF and, therefore, vascular proliferation^2,6,24^.

The appearance of IOALs can vary on different imaging modalities based on the degree of fat within them. They appear as radiolucent lesions on X-ray, and often demonstrate low attenuation on computed tomography images with Hounsfield number measurements ranging from −80 to +78^10,18^. Nearly two-thirds of IOALs were expansile^2–4,6–11,13,15,19,21^. On MRI, they exhibit variable intensity on T1-weighted images and are hyperintense on T2-weighted images, and isointense on PD-weighted images, with the signal from the fatty component being properly suppressed on fat-suppression sequences (e.g. Fat-Sat and STIR).

Scintigraphy is of no diagnostic value in diagnosing IOALs, but can help differentiate meningiomas from cranial IOALs^11^.

Many differential diagnoses of IOALs exist and should be taken into consideration, such as aneurysmal bone cysts, lipomas, angiomas, hemangiomas, giant cell tumors, fibrous dysplasia, and meningiomas. These could be ruled out depending on their radiological features and histopathological findings.

Surgical excision is the primary treatment option, with embolization being a viable alternative in some cases^3,4,6^.

## Conclusion

In conclusion, it is difficult to accurately diagnose an IOAL, which is why careful evaluation and planning are essential to ensure proper management of the lesion. Histological findings are key to making a definite diagnosis, and surgery is typically the preferred treatment option.

## Ethical approval

This case report did not require review by the ethics committee in Aleppo University Hospital (AUH), Aleppo, Syria.

## Consent

Written informed consent was obtained from the patient for the publication of this case report and accompanying images. A copy of the written consent is available for review by the Editor-in-Chief of this journal on request.

## Sources of funding

This research did not receive any specific grant from funding agencies in the public, commercial, or not-for-profit sectors.

## Author contribution

F.K.K.: contributed to data collection and interpretation, study concept and design, and writing the paper (first author and corresponding author); S.K.: contributed to data collection and interpretation and writing the paper (coauthor #1); S.S.T.: contributed to data collection and interpretation, study concept and design, and writing the paper (coauthor #2); M.W.A.: contributed in data collection and reviewing the paper (coauthor #3); M.I.E.M.: contributed in data collection and interpretation (coauthor #4).

## Conflicts of interest disclosure

All authors declared that there are no conflicts of interest.

## Research registration unique identifying number (UIN)

Not applicable.

## Guarantor

Firas K. Khana.

## Provenance and peer review

Not commissioned; externally peer-reviewed.
